# Patient involvement in research – participants or collaborators?

**DOI:** 10.1111/hex.12578

**Published:** 2017-05-16

**Authors:** Carolyn A. Chew‐Graham

**Affiliations:** ^1^ Primary Care and Health SciencesKeele UniversityStaffordshireST5 5BGUK

There is a consensus that the public should be involved in health research, and Patient and Public Involvement and Engagement (PPIE) can be at many different stages of research.^1^ In this edition of HEX, Blackburn's letter outlines the difference between patients as participants in research (referring to a manuscript outlining the role of patients establishing the content of a patient‐reported outcome measure^2^) versus a role as an active participant in the whole research process. His comments are helpful in illuminating the dilemma which the Editors at HEX often have in handling submitted manuscripts.

Our website outlines the range of manuscripts that we would like to publish (Box 1), and states that the editors require authors to include a description of any significant patient involvement in one or more of the following research processes: selecting and agreeing the research question; study design and methods; interpretation and discussion of the study findings; and dissemination of results.

Box 1 Submitted manuscripts to *Health Expectations* should describe involvement of:

- patients and their advocates in decisions about individual health care;
- health service users and their representatives in aspects of service design, delivery and evaluation;
- health service users and family members in efforts to enhance the quality and safety of care;
- the wider public in debates about health‐care policy.

In this edition, Mantovani and colleagues report a qualitative study where people were informants in a qualitative study, but the very nuanced analysis, the editors felt, means that this manuscript has important implications for commissioning. Mantovani and colleagues suggest that their study will contribute to the delivery of culturally sensitive mental health care and services which is more congruent with the needs of diverse populations.

Crocker and colleagues report their qualitative study exploring patient and public involvement (PPI) in health research, identifying a range of roles that they feel PPI contributors can play: the ‘lived experience’ role echoes qualitative studies such as Mantovani's. Crocker's labels for roles in which PPI reflects a collaborative approach include the ‘creative insider’, the ‘free challenger’, the ‘bridger’ or the ‘motivator’. We would welcome submissions in which PPIE reflects these more complex roles.

Two papers report the role of patients in improving the quality of health services. Thus, Wright and colleagues report the results of an exploratory trial of real‐time feedback (RTF), which involves collecting and summarizing information about patient experience at the point of care, with the aim of informing service improvement. They highlighted that only 2.5% of consulting patients provided feedback, although patients who did were broadly positive about the concept of RTF. Savia de Souza and colleagues describe how patients can effectively contribute to service improvement provided they are supported, respected as equals, and the organization is willing to undergo a cultural change.

O'Shea and colleagues report a qualitative study which explored the lay role in one clinical commissioning group (CCG), highlighting a continued lack of clarity about roles, and consequent lack of impact on decision making. Clearly more work is needed in making the public role in CCGs more meaningful.

Finally, we are pleased to welcome two new members to the Editorial team: Parisi Aslani and Louise Condon have joined HEX as Associate Editors.

Professor Parisi Aslani is Professor in Medicines Use Optimisation at the University of Sydney, where she has both teaching and research roles. She brings broad expertise in research methods, PPIE and has experience in reviewing for a broad range of journals. Professor Louise Condon has a community nursing and midwifery background, and is very involved in Public Policy and Education. Louise is Professor at the College of Human and Health Sciences at Swansea University, UK. Prior to taking up the Associate Editor role, Professor Condon was an active member of the HEX Editorial Board.

These two excellent appointments should ensure the continued high quality and speedy reviewing of manuscripts submitted to HEX.
